# Role of the New Physical Examination Minor Criterion (New Heart Murmur) for the Diagnosis of Infective Endocarditis

**DOI:** 10.1093/ofid/ofae736

**Published:** 2024-12-19

**Authors:** Matthaios Papadimitriou-Olivgeris, Pierre Monney, Michelle Frank, Georgios Tzimas, Nicolas Fourré, Virgile Zimmermann, Piergiorgio Tozzi, Matthias Kirsch, Mathias Van Hemelrijck, Omer Dzemali, Jana Epprecht, Benoit Guery, Barbara Hasse

**Affiliations:** Infectious Diseases Service, Lausanne University Hospital and University of Lausanne, Lausanne, Switzerland; Infectious Diseases Service, Cantonal Hospital of Sion and Institut Central des Hôpitaux, Sion, Switzerland; Department of Cardiology, Lausanne University Hospital and University of Lausanne, Lausanne, Switzerland; Department of Cardiology, University Hospital Zurich and University of Zurich, Zurich, Switzerland; Department of Cardiology, Lausanne University Hospital and University of Lausanne, Lausanne, Switzerland; Infectious Diseases Service, Lausanne University Hospital and University of Lausanne, Lausanne, Switzerland; Infectious Diseases Service, Lausanne University Hospital and University of Lausanne, Lausanne, Switzerland; Department of Cardiac Surgery, Lausanne University Hospital and University of Lausanne, Lausanne, Switzerland; Department of Cardiac Surgery, Lausanne University Hospital and University of Lausanne, Lausanne, Switzerland; Department of Cardiac Surgery, University Hospital Zurich and University of Zurich, Zurich, Switzerland; Department of Cardiac Surgery, University Hospital Zurich and University of Zurich, Zurich, Switzerland; Center for Translational and Experimental Cardiology, Department of Cardiology, University Hospital Zurich and University of Zurich, Zurich, Switzerland; Department of Infectious Diseases and Hospital Epidemiology, University Hospital Zurich and University of Zurich, Zurich, Switzerland; Infectious Diseases Service, Lausanne University Hospital and University of Lausanne, Lausanne, Switzerland; Department of Infectious Diseases and Hospital Epidemiology, University Hospital Zurich and University of Zurich, Zurich, Switzerland

**Keywords:** Duke criteria, heart murmur, infective endocarditis, International Society for Cardiovascular Infectious Diseases (ISCVID), physical examination

## Abstract

Among 3127 episodes of suspected infective endocarditis, the 2023 Duke–International Society for Cardiovascular Infectious Diseases clinical criteria showed an accuracy of 90% for infective endocarditis diagnosis. A new heart murmur was present in 690 (22%) episodes. Excluding imaging and surgical findings decreased the accuracy to 73%, while using the physical examination criterion slightly improved the accuracy to 78%.

## Teaser

Among 3127 episodes of suspected infective endocarditis, the 2023 Duke–International Society for Cardiovascular Infectious Diseases clinical criteria showed low accuracy (78%) when excluding the imaging and surgical findings and when solely using the physical examination criterion.

Infective endocarditis (IE) remains a diagnostic challenge due to its variable clinical presentation. The primary diagnostic tool is cardiac imaging, with transthoracic echocardiography and transesophageal echocardiography (TEE) serving as the first-line imaging modalities [1]. Over the past decade, additional imaging techniques, such as ^18^F-fluorodeoxyglucose positron emission tomography/computed tomography (^18^F-FDG PET/CT) and cardiac CT, have proven valuable, particularly for complex cases where echocardiography is inconclusive, TEE is not feasible, or prosthetic valve IE is suspected [1]. These imaging modalities are now integral to the diagnostic algorithms proposed by the European Society of Cardiology (ESC), highlighting their importance [1]. Their findings are also included in the major imaging criteria of the two 2023 versions of the Duke criteria, one from the ESC and the other from the International Society for Cardiovascular Infectious Diseases (ISCVID) [1, 2], both of them showing an increase in sensitivity as compared with the 2015 ESC version [3–6].

Despite the importance of these imaging modalities, their availability varies significantly by region [7, 8]. In the EURO-ENDO cohort, TEE was performed on 24% of patients with IE in the Middle East and 30% in South Asia, as opposed to 66% in Western Europe and 53% in Southern Europe [7]. An international survey of 2031 physicians revealed that while ^18^F-FDG PET/CT was available to 78% of the respondents in Europe, it was accessible to only 9% in Africa [8]. This disparity in diagnostic resources has led to the inclusion of a new minor physical examination criterion in the ISCVID version of the Duke criteria, based on the presence of a new heart murmur [2]. This criterion, included per expert opinion, should be applied only if imaging modalities are unavailable [2].

This study aimed to evaluate the performance of the 2023 Duke-ISCVID clinical criteria, with and without the new minor physical examination criterion, in a multicenter cohort of patients with suspected IE, excluding cardiac imaging and surgical findings [9].

## METHODS

This study, conducted at Lausanne University Hospital (CHUV) and University Hospital Zurich (USZ) in Switzerland, combined 3 cohorts: a retrospective/prospective cohort of patients with suspected IE from CHUV (January 2014–June 2023; prospective inclusion from 2018 onward), a retrospective cohort of patients with bacteremia/candidemia from CHUV (January 2015–December 2021), and a retrospective/prospective cohort of patients with IE from USZ (January 2014–December 2022; prospective inclusion from 2018 onward). Ethical approval was obtained from Swiss Ethics Committees (CER-VD-2017-02137, CER-VD-2021-02516; KEK-2014-0461; BASEC-2017-01140).

Eligible participants included adult patients (≥18 years) suspected of having IE, defined as those who had blood cultures drawn and echocardiography performed specifically for IE diagnosis. Additional criteria included no data refusal for the retrospective cohort and written consent for the prospective cohort.

In both centers, a bedside infectious diseases consultation is mandatory for all suspected IE cases. Since 2018, endocarditis team at each center have classified patients as having IE. Prior to 2018, 5 expert clinicians (CHUV: M.P.-O., P.M.; USZ: M.F., M.V.H., B.H.) assessed and classified each case as rejected, possible, or definite IE using the 2023 Duke-ISCVID clinical criteria [2]. We applied 3 sets of classifications: the first set used the full Duke-ISCVID criteria; the second set excluded major and minor imaging criteria as well as the major surgical criterion; and the third set applied the minor physical examination criterion. The variable physical examination criterion was defined as any new heart murmur in patients without a preexisting murmur or those with no prior physical examination. The 2 CHUV cohorts collect data on preexisting heart murmurs.

We used SPSS version 26.0 (IBM) for the statistical analysis. We used the Mann-Whitney *U* test to assess continuous variables, while the χ^2^ [2] or Fisher exact test was employed for examining categorical variables. To evaluate the performance of the different iterations of the 2023 Duke-ISCVID clinical criteria, we compared diagnoses provided by the endocarditis teams or the expert clinicians (considered the reference standard) with cases identified as definite IE based on these criterion iterations. We calculated sensitivity, specificity, positive and negative predictive values, and accuracy with the corresponding 95% CI in the entire cohort. All tests were 2-tailed, and *P* < .05 was considered statistically significant.

## RESULTS

Among the 3127 episodes of suspected IE from all 3 cohorts, 1177 (38%) were diagnosed with IE by the endocarditis teams or expert clinicians. Thereof, 752 (64%) were linked to native valves, 317 (27%) to prosthetic valves, 180 (15%) to cardiac implantable electronic device leads, and 7 (0.6%) to other intracardiac structures. Transthoracic echocardiography was performed in 2822 (90%) episodes, TEE in 1494 (48%), ^18^F-FDG PET/CT in 468 (15%), and cardiac CT in 84 (3%). The major imaging criterion was present in 987 (32%) episodes (Table 1), occurring more frequently in IE episodes (79% vs 3%, *P* < .001). Valve surgery was performed in 479 (15%) episodes, with 235 (49%) showing macroscopic evidence of IE. Physical examination revealed a new heart murmur in 690 (22%) episodes, which was more common in patients with IE than those without (37% vs 13%, *P* < .001). Among the 2618 episodes from the 2 CHUV cohorts, 802 (31%) had a murmur (new or preexisting), with a higher rate among patients with IE (56% vs 22%, *P* < .001).

**Table 1.** Episodes With and Without Infective Endocarditis Diagnosis Among 3127 Patients With Suspected Infective Endocarditis

	Infective Endocarditis, No. (%)		
	No (n = 1950)	Yes (n = 1177)	*P* Value
Male sex	1329 (68)	879 (75)	<.001
Age, y, median (IQR)	68 (55–78)	66 (51–75)	<.001
Major criterion			
Microbiological	835 (43)	946 (80)	<.001
Imaging	52 (3)	935 (79)	<.001
Surgical	0 (0)	8 (0.7)	<.001
Minor criterion			
Predisposition	524 (26)	872 (74)	<.001
Fever	1548 (79)	920 (78)	.416
Vascular	210 (11)	622 (53)	<.001
Immunologic	27 (1)	149 (13)	<.001
Microbiological	320 (16)	80 (7)	<.001
Classification according to ISCVID clinical criteria			<.001
Rejected	1056 (54)	35 (3)	
Possible	866 (44)	262 (22)	
Definite	28 (1)	880 (75)	
Classification according to ISCVID clinical criteria without imaging and surgical criterion			<.001
Rejected	1094 (56)	156 (13)	
Possible	840 (43)	659 (56)	
Definite	16 (0.8)	362 (31)	
Minor physical examination criterion	260 (13)	430 (37)	<.001
Classification according to ISCVID clinical criteria without imaging and surgical criterion and with the new minor physical examination criterion			<.001
Rejected	1037 (53)	124 (11)	
Possible	858 (44)	524 (45)	
Definite	55 (3)	529 (45)	

Abbreviation: ISCVID, International Society for Cardiovascular Infectious Diseases.

The 2023 Duke-ISCVID clinical criteria demonstrated an accuracy of 90% (95% CI, 88%–99%) for IE diagnosis. Excluding imaging/surgical findings decreased the accuracy to 73% (95% CI, 71%–75%), while using the physical examination criterion improved the diagnostic accuracy only slightly (78% [95% CI, 76%–79%]; Table 2).

**Table 2.** Performance of the 2023 Duke-ISCVID Clinical Criteria Among 3127 Episodes With Suspected Infective Endocarditis With and Without the New Murmur Criterion

	% (95% CI)				
	Sensitivity	Specificity	PPV	NPV	Accuracy
**2023 Duke-ISCVID**	75 (72–77)	99 (98–99)	97 (96–98)	87 (85–88)	90 (88–91)
Without imaging/surgical criterion	31 (28–33)	99 (99–100)	96 (93–97)	70 (70–71)	73 (72–75)
Without imaging/surgical criterion with the physical examination criterion	45 (42–48)	97 (96–98)	91 (88–93)	75 (74–76)	78 (76–79)

Abbreviations: ISCVID, International Society for Cardiovascular Infectious Diseases; NPV, negative predictive value; PPV, positive predictive value.

## DISCUSSION

In this study, we found that the diagnostic performance of the 2023 Duke-ISCVID clinical criteria was poor when imaging and surgical findings were excluded (accuracy of 73%), even when using the minor physical examination criterion (accuracy of 78%).

The ISCVID recommended including a minor physical examination criterion, to be used only when cardiac imaging examinations are unavailable [2]. This addition aimed to assist centers with limited resources in diagnosing IE [7]. While applying this criterion did increase sensitivity (45% vs 34% without), the sensitivity remained too low for safe clinical application. This not only highlights the essential role of cardiac imaging in IE diagnosis but also affirms the importance of imaging modalities as a part of the major criteria alongside microbiological findings [1, 2]. Therefore, we propose that the ISCVID remove the minor physical examination criterion in the next iteration of the criteria. Additionally, the variability in identifying heart murmurs presents a significant challenge to using this criterion within strict diagnostic frameworks [10]. Any heart murmur (new or preexisting) was present in 56% of episodes, comparable to the findings in the EURO-ENDO cohort and the International Collaboration on Endocarditis–Prospective Cohort Study, with 65% and 68% of patients, respectively [7, 11]. The rate of new heart murmurs in patients with IE (37%) was comparable to the GAMES cohort (32%) and the International Collaboration on Endocarditis–Prospective Cohort Study (48%) [11, 12].

With the same cohort, the 2023 Duke-ISCVID criteria showed increased sensitivity (76%) as compared with the Duke-ESC clinical criteria of 2015 (59%) and 2023 (69%) [9]. While there are differences between the 2023 Duke-ISCVID and 2023 Duke-ESC criteria—such as the addition of leaflet thickening to the major imaging criterion and hematogenous osteoarticular septic complications to the minor vascular phenomenon criterion by the latter—the better performance of the 2023 Duke-ISCVID criteria is attributed to the comprehensive adaptation of microorganisms considered typical for IE [1, 2, 9]. Studies evaluating the 2023 Duke-ISCVID and 2023 Duke-ESC criteria have shown conflicting results [3–5]. One study focusing on patients with suspected IE and another on patients with *Staphylococcus aureus* bacteremia found comparable sensitivities between the versions [3, 4]. However, a study based on patients with streptococcal bacteremia found that the 2023 Duke-ISCVID criteria had higher sensitivity than the 2023 Duke-ESC criteria [5].

Our study has several limitations. First, the majority of patients were included retrospectively. Additionally, the study was conducted in only 2 tertiary centers in a high-resource country with direct access to advanced cardiac imaging modalities, which may limit the applicability of our findings to other health care settings. Furthermore, the lack of a definitive gold standard for IE diagnosis could lead to misclassification of episodes. To address this limitation, we used the classification provided by the endocarditis teams and expert clinicians. Finally, the detection of heart murmurs may be influenced by the auscultation skills of the treating physician [10], although this bias was mitigated by the fact that in both centers, all patients with suspected IE were seen by infectious diseases physicians.

In conclusion, the 2023 Duke-ISCVID clinical criteria should not be used to diagnosis IE in the absence of cardiac imaging, even when the physical examination minor criterion is used. Instead, the international medical community should focus on increasing the availability of cardiac imaging techniques, particularly echocardiography, in resource-limited settings.

## Notes

***Author contributions*.** B. H. and M. P.-O. conceived the idea. M. P.-O., P. M., M. F., G. T., N. F., V. Z., P. T., M. K., M. V. H., O. D., J. E., B. G., and B. H. collected the patients’ data. B. H. and M. P.-O. supervised the project. M. P.-O. and B. H. performed the analysis. All authors interpreted the results. M. P.-O. wrote the first draft of the manuscript. All authors contributed to manuscript revision and read and approved the submitted version.

***Financial support*.** This work was supported by the Swiss National Science Foundation (grants 32003B_219351/1 to B. H. and M. P.-O. and 320030_184918/1 to B. H.); and the Clinical Research Priority Program of the University of Zurich for the Clinical Research Priority Program Precision medicine for bacterial infections (to B. H.).

## References

[1] Delgado V, Ajmone Marsan N, de Waha S, et al 2023 ESC guidelines for the management of endocarditis. Eur Heart J 2023; 44:3948–4042. 37622656 10.1093/eurheartj/ehad193

[2] Fowler VG, Durack DT, Selton-Suty C, et al The 2023 Duke-ISCVID criteria for infective endocarditis: updating the modified Duke criteria. Clin Infect Dis 2023; 77:518–26. 37138445 10.1093/cid/ciad271 PMC10681650

[3] van der Vaart TW, Bossuyt PMM, Durack DT, et al External validation of the 2023 Duke—International Society for Cardiovascular Infectious Diseases diagnostic criteria for infective endocarditis. Clin Infect Dis 2024; 78:922–9. 38330166 10.1093/cid/ciae033 PMC11006110

[4] Papadimitriou-Olivgeris M, Monney P, Frank M, et al Evaluation of the 2023 Duke-ISCVID and 2023 Duke-ESC clinical criteria for the diagnosis of infective endocarditis in a multicenter cohort of patients with *Staphylococcus aureus* bacteremia. Clin Infect Dis 2024; 78:655–62. 38168726 10.1093/cid/ciae003 PMC10954331

[5] Fourre N, Zimmermann V, Senn L, et al Evaluation of the HANDOC score and the 2023 ISCVID and ESC Duke clinical criteria for the diagnosis of infective endocarditis among patients with streptococcal bacteremia. Clin Infect Dis 2024; 79:434–42. 38842414 10.1093/cid/ciae315 PMC11327781

[6] Papadimitriou-Olivgeris M, Monney P, Frank M, et al Evaluation of the 2023 Duke–International Society of Cardiovascular Infectious Diseases criteria in a multicenter cohort of patients with suspected infective endocarditis. Clin Infect Dis 2024; 78:949–55. 38330243 10.1093/cid/ciae039 PMC11006096

[7] Habib G, Erba PA, Iung B, et al Clinical presentation, aetiology and outcome of infective endocarditis. Results of the ESC-EORP EURO-ENDO (European infective endocarditis) registry: a prospective cohort study. Eur Heart J 2019; 40:3222–32. 31504413 10.1093/eurheartj/ehz620

[8] Westgeest AC, Buis DTP, Sigaloff KCE, et al Global differences in the management of *Staphylococcus aureus* bacteremia: no international standard of care. Clin Infect Dis 2023; 77:1092–101. 37310693 10.1093/cid/ciad363 PMC10573727

[9] Papadimitriou-Olivgeris M, Monney P, Frank M, et al Comparison of the 2015 and 2023 European Society of Cardiology versions of the Duke criteria among patients with suspected infective endocarditis. Clin Infect Dis. Published online 12 July 2024. doi:10.1093/cid/ciae370 PMC12043060 38997115

[10] Codispoti CA, Eckart RE, Rutberg SA, Shry EA, Boyd SY. Appreciation of precordial cardiac murmur on examination relative to knowledge of valvular heart disease. Cardiol Rev 2005; 13:147–51. 15831149 10.1097/01.crd.0000134916.93707.d0

[11] Murdoch DR, Corey GR, Hoen B, et al Clinical presentation, etiology, and outcome of infective endocarditis in the 21st century: the International Collaboration on Endocarditis–Prospective Cohort Study. Arch Intern Med 2009; 169:463–73. 19273776 10.1001/archinternmed.2008.603 PMC3625651

[12] Munoz P, Kestler M, De Alarcon A, et al Current epidemiology and outcome of infective endocarditis: a multicenter, prospective, cohort study. Medicine (Baltimore) 2015; 94:e1816. 26512582 10.1097/MD.0000000000001816 PMC4985396

